# Referee acknowledgement for 2019

**DOI:** 10.1038/s41416-020-0880-0

**Published:** 2020-07-02

In this issue, we publish the names of those who reviewed manuscripts for us in 2019. The Editor-in-Chief, Subject Editors and everyone involved in publishing *BJC* would like to extend our sincere thanks to the referees for contributing their expertise and time.

- Reza Abdi
- Abhinav Achreja
- Helmut Acker
- Peter D Adams
- Sarah Adams
- Monika Aggarwal
- Po Agnese
- Pedro Aguiar Jr
- Thomas Ahern
- Aamir Ahmad
- Imran Ahmad
- Nihal Ahmad
- Mansoor Ahmed
- Samreen Ahmed
- Sam Hjelmeland Ahmedzai
- Joong Bae Ahn
- Iannis Aifantis
- Ioannis Aifantis
- Driss Ait Ouakrim
- Guray Akturk
- Giovanni Aletti
- Alain P Algazi
- H Raza Ali
- Simak Ali
- Malcolm R Alison
- Fabrice Allain
- James Allan
- Alhadi Almangush
- R Almeida
- Raquel Almeida
- Abdo Alnabulsi
- Barbara Altieri
- Marianna Alunni-Fabbroni
- Frederic Amant
- Peter F Ambros
- Antonio Amelio
- Christopher I Amos
- Dimitrios Anastasiou
- Chelsea Anderson
- Jana Jane Anderson
- Mary Anderson
- Anne Andersson
- Kartik Angara
- Alan Anthoney
- Thomas Aparicio
- Andrew E Aplin
- Marc Arbyn
- Takaaki Arigami
- Jane M Armer
- Jane Armes
- Melina Arnold
- Rudolf Arnold
- Melyssa Aronson
- Eric Assenat
- Chantal Autexier
- Philippe Autier
- Karol Axcrona
- Nagi Ayad
- Duncan Ayers
- Laurent Azoulay
- Stefan Böhringer
- Ren-Yuan Ba
- Hideo Baba
- Jean-Baptiste Bachet
- Sunil S Badve
- Rajendra Badwe
- Zsuzsanna Bago-Horvath
- Mingfeng Bai
- Andrei Bakin
- Gustavo Baldassarre
- Tejus A Bale
- Alberto Ballestrero
- Fatima Baltazar
- Rajkumar Banerjee
- Sulagna Banerjee
- Udai Banerji
- Gaspar Banfalvi
- Eli Bar
- Gil Bar-Sela
- Andrea Baragetti
- Alan W Barclay
- Matthew E Barclay
- Jorge Barriuso
- Desmond P Barton
- Bristi Basu
- Peter Bay
- Stephen Baylin
- Martina Bazzaro
- Carlos Becerra
- Jůrgen Becker
- Paul Beckett
- Muhammad Shaalan Beg
- Andrew D Beggs
- Fariba Behbod
- Carmen Behrens
- Tanios Bekaii-Saab
- Mattias Belting
- Par-Ola Bendahl
- Christina D Berg
- Martin R Berger
- Walter Berger
- Anna Sophie Berghoff
- Johannes Berkhof
- Carmelo Bernabéu
- Alfredo Berruti
- Anna Berthel
- Frank Berthold
- Joseph R Bertino
- Francesco Bertolini
- Giulia Bertolini
- Anamika Bhargava
- Rajat Bhattacharya
- Shree Bhide
- Roberto Bianco
- Andrew V Biankin
- Alan Bilsland
- Ranjit Bindra
- Justin A Bishop
- Sarah P Blagden
- Mikhail V Blagosklonny
- Jean-Yves Blay
- Jeremy Blaydes
- Carl Blomqvist
- C Richard Boland
- Patrick M Boland
- Hervé Bonnefoi
- Michael A Bookman
- Domenico Borzomati
- FT Bosman
- Stefan Bosner
- Dominick Bosse
- Jan Bouchal
- Katerina Bouchalova
- Yves Boucher
- Ioannis Boukovinas
- Jean-Christophe Bourdon
- Ben Boursi
- Chiara Braconi
- Gerard Brady
- Simone Braig
- Michael Graham Bramble
- Jesper Bertram Bramsen
- Petter Brandal
- Sebastian Brandner
- Isabel Bravo
- Nancy Breen
- Paul Brennan
- Hermann Brenner
- John Chad Brenner
- Adam Robert Brentnall
- John Bridgewater
- Sara Briggs
- Philip James Broadhurst
- Mireille JM Broeders
- Stefan Broeer
- Ewan Brown
- J Martin Brown
- Janet Brown
- Robert Brown
- Adam Brufsky
- Paal Fr Brunsvig
- Richard J Bryant
- Niamh E Buckley
- Ronald M Bukowski
- Carlo Buonerba
- Nikolaos Burbos
- Andrea Buron
- Ovidio Bussolati
- Francesco Bussu
- Marc Buyse
- Gemma Cadby
- Guoxiang Cai
- Qiliang Cai
- Sanjun Cai
- Saverio Caini
- Philip Calder
- George Calin
- Anna Calio
- Grace Callagy
- David Cameron
- Ian Campbell
- Kerry Campbell
- William Cance
- Amparo Cano
- Yihai Cao
- Michele Carbone
- Timothy Card
- Elyce Cardonick
- Ross Carruthers
- Louise Carter
- Richard Carvajal
- Andrea Casadei-Gardini
- Ignacio Casal
- Angela Casbard
- Philippe Alexandre Cassier
- Maria Castro
- Guido Cavaletti
- Stefano Cavalieri
- Kishore B Challagundla
- Arlene Chan
- Jefferson Y Chan
- June MayLin Chan
- Dhyan Chandra
- Joya Chandra
- Manju Chandrasegaram
- Stephen J Chanock
- Lorraine Chantrill
- Michel Charbonneau
- Hadrien Charvat
- Greg Charville
- Georgia Chen
- Lingchao Chen
- Rong-Xin Chen
- Shaoyong Chen
- Bin Cheng
- Chao Cheng
- Xiang Cheng
- John Chester
- Paola Chiarugi
- Mathieu Chicard
- Mariona Chicon
- Kazuaki Chikamatsu
- Pierce KH Chow
- Brock C Christensen
- Alan Christie
- Liz Christie
- A Jung Chu
- Yuen-Li Chung
- David Church
- Ashley Cimino-Mathews
- Kristen K Ciombor
- Maria Rosa Ciriolo
- Eva Ciruelos
- Ludovica Ciuffreda
- Brian Clark
- Hamish Clouston
- Lan Coffman
- Graham Colditz
- Kimberly Cole
- Helen Coleman
- Eva Coll De la Rubia
- Graham Peter Collins
- Peter Collins
- Fiona Collinson
- Luca Colucci-D’Amato
- Barbara Conley
- Neil Conlon
- James L Connolly
- Marcus Conrad
- PierFranco Conte
- Gordon Cook
- Natalie Cook
- Colin Cooper
- Mhairi Copland
- Laurent Corcos
- Michelangelo Cordenonsi
- Alex Cornish
- Maria Valeria Corrias
- Pippa G Corrie
- Victoria Cortessis
- Casey Cosgrove
- Julia Costa
- Tricia Cottrell
- Karina Cox
- Danielle Crawley
- Ian A Cree
- Nicola Cresti
- Lucio Crinò
- Carmen Criscitiello
- Susan Critchlow
- William Cross
- Alessandro Cucchetti
- Zoran Culig
- Giuseppe Curigliano
- Kate Cuschieri
- Brian G Czito
- Simona D’Aguanno
- Andre D’Hoore
- Anneleen Daemen
- Kristina R Dahlstrom
- Wei Dai
- Angus G Dalgleish
- Blossom Damania
- Chendil Damodaran
- Sarah Danson
- Kaustubh Datta
- David W Dawson
- Colin Dayan
- Dario De Biase
- Filippo de Braud
- Anna de Fazio
- Ugo De Giorgi
- Steven de Jong
- Harry J de Koning
- Astrid Marie De Meulenaere
- Theo M de Reijke
- Anita De Rossi
- Emma De Ruiter
- Carmela De Santo
- Hugo De Vuyst
- L DeAngelis
- Thomas Decaens
- Cristina Dechecchi
- Shoukat Dedhar
- Suha Deen
- Hadassa Degani
- Scott M Dehm
- Paul Delrée
- Paolo Delrio
- Carla DeMuro
- Tusar K Desai
- Giovan Giuseppe Di Costanzo
- Massimo Di Maio
- Massimo Di Nicola
- Julie Di Paolo
- Maria Flavia Di Renzo
- Gabriela Di Venosa
- Dolores Di Vizio
- Robert B Diasio
- Mario Dicato
- Paul W Dickman
- Veronika Dill
- Richard Dillon
- Jian Ding
- Wan-Jun Ding
- Luc Yves Dirix
- David Dodwell
- Eva Dombi
- Joseph Domingo-Domenech
- Jixin Dong
- Linda Dong
- Tanya Dorff
- Joanne F Dorgan
- Chyke A Doubeni
- Antonios Douros
- Amy Downing
- Mitch Dowsett
- Charles W Drescher
- Yvette Drew
- Catherine Dube
- Ludwig J Dubois
- Yuri Dubrova
- Diana Dudziak
- Stephen W Duffy
- Nancy Dumont
- Elaine Dunlop
- Malcolm Dunlop
- Raul Duran
- Steve Durant
- Uma Duvvuri
- Ben Dwamena
- Martin Dyer
- Paul J Dyson
- Angela B Edgar
- Nina Frederike Jeppesen Edin
- Thomas Efferth
- Alexander Egle
- Bent Ejlertsen
- Imane El-Dika
- Mohamad Elbaz
- Elena Elez Fernandez
- Kevin M Elias
- Aristides Eliopoulos
- Matthew J Ellis
- Peter Ellis
- Anneli Elme
- Kevin H Eng
- Christine Engeland
- Mathew Erhardt
- Mikael Eriksson
- Fiona Errington
- Blanca Espinet
- Irene Esteban
- Manel Esteller
- D Gareth Evans
- Par Ewing
- Anniina Färkkilä
- Stefano Fais
- Marco Falasca Falasca
- Stephen J Falk
- Meiyun Fan
- Weiyi Fang
- Ruth Farmer
- Susan Mary Farrington
- Peter Fasching
- Matteo Fassan
- Waleed Fateen
- Inna Fedorenko
- Elizabeth Lieke AM Feijen
- Seth Felder
- Klaus Felix
- Emanuela Felley-Bosco
- Weiwei Feng
- Dyke Ferber
- Mårten Fernö
- Elisabetta Ferretti
- Anna Fialova
- William Figg
- Victoria J Findlay
- David Fine
- Stefania Fiorcari
- Giammaria Fiorentini
- Michael Fisher
- Paul B Fisher
- Marie-Louise Fjällskog
- Robert Flavell
- Angela Fong
- S Lie Fong
- Andrea Forschner
- Renée Turzanski Fortner
- Even Fossum
- David A Foster
- Christina Fotopoulou
- Michael Fradley
- Hester Franks
- Callum G Fraser
- Henrik Frederiksen
- Hanna Fredholm
- Neal D Freedman
- Jose Freije
- Heinz Freisling
- CA French
- Rafael Fridman
- Wolf Herve Fridman
- Christine M Friedenreich
- Susan Frost
- Robert Fruscio
- Yang-Xin Fu
- Takaaki Fujii
- Masashi Fukayama
- Ben Fulton
- Hans-Joachim Gabius
- Christian Gaiddon
- Udo Gaipl
- Francoise Galateau-Salle
- Ferdia Gallagher
- Paolo Gallipoli
- Ram N Ganapathi
- Q Gao
- Yuzhen Gao
- Antonio García de Herreros
- Alejandro García-Carrancá
- Pilar Garcia Alfonso
- Montserrat Garcia-Closas
- Michela Garofalo
- Elena Garralda
- Dale W Garsed
- Mark Garzotta
- Don M Gash
- Nathalie Gaspar
- Michael Gatza
- Andrew Gaya
- Yubin Ge
- Sandra J Gendler
- Suzanne George
- Vassilis Georgoulias
- Marco Gerlinger
- Grigoris T Gerotziafas
- Timothy Gershon
- Francois Ghiringhelli
- Paramita M Ghosh
- Giuseppe Giaccone
- Marios Giannakis
- Ziv Gil
- Duncan C Gilbert
- Ethel S Gilbert
- Philipp Gild
- Carolynn Gildea
- Sharlene Gill
- Robert Gillies
- Roopinder Gillmore
- Daniel Gioeli
- Thomas J Giordano
- Edward L Giovannucci
- A Girard
- Sandhya Girish
- Aleksander Giwercman
- Dylan Glubb
- Sanjay Goel
- Michael Goggins
- James J Going
- Torsten Goldmann
- Sandra O Gollnick
- Roger Gomis
- Aaron Goodman
- Sharon Gorski
- Yasushi Goto
- Noriko Gotoh
- Ramaswamy Govindan
- David Graham
- George D Grass
- Jaume Grau
- Kristi Graves
- Mel Greaves
- William Greenhalf
- Alastair Greystoke
- Arjan W Griffioen
- Gaia Griguolo
- Alessandro Gronchi
- Isabelle Gross
- Ashley Grossman
- Shilpa Grover
- Richard G Grundy
- Thomas W Grunt
- Guowei Gu
- Greta Guarda
- Jennifer Guerriero
- Fabienne Guillaumond
- Gilles J Guillemin
- Deliang Guo
- Junming Guo
- Marianne Grønlie Guren
- Alejandro Gutierrez
- Bastian Höchst
- Michael Haas
- Nikolas K Haass
- Nadia Haddy
- Elin Hadler-Olsen
- Christel Haggstrom
- Hanne R Hagland
- Pierre Hainaut
- Bengt Hallberg
- Balazs Halmos
- Akinobu Hamada
- Robert Hamanaka
- Michael R Hamblin
- Petra Hamerlik
- Wonshik Han
- Philip C Hannaford
- Aaron R Hansen
- Torben Frøstrup Hansen
- Reina Haque
- Mamoru Harada
- Arja Harila-Saari
- Joshua Chuck Harrell
- Stefan Hartmann
- Koichiro Haruki
- Maryam Hashemian
- Matthew Q Hatton
- Lance Heilbrun
- Florian Heitz
- Nadine Hempel
- Shona Hendry
- S Jane Henley
- S Henri
- Alexander G Heriot
- Patries Herst
- Antony Hickey
- Blánaid Hicks
- David Hill
- Samantha Hinsley
- Kevin Hiom
- Nobuyoshi Hiraoka
- Angela Hirbe
- Thomas K Hoffmann
- Ralf-Dieter Hofheinz
- Wolfgang Hohenforst-Schmidt
- Antoine Hollebecque
- Lars Holmberg
- Jeff Holst
- David S Hong
- Nicoline Hoogerbrugge
- Lisa G Horvath
- Peter J Hoskin
- Sebastian Hotte
- Courtney Houchen
- Meichin Hsieh
- Po-Kuei Hsu
- Chang-Deng Hu
- Dong Hua
- P Huang
- Qiaojia Huang
- Qichao Huang
- Mike Hubank
- Peter E Huber
- Robert A Huddart
- Alzbeta Hulikova
- Kent W Hunter
- Paula Hurley
- Chris Hydes
- Torukiri I Ibiebele
- David H Ilson
- Mohammad Ilyas
- Seock-Ah Im
- Stefano Indraccolo
- SP Ingram
- Julie Irving
- C Isacke
- Genichiro Ishii
- Hiroki Ishii
- Takashi Ishikawa
- Takatsugu Ishimoto
- Alexander Ishov
- Roohi Ismail-Khan
- Jorma Isola
- Michael Ittmann
- Mircea Ivan
- Takayuki Iwamoto
- Masaaki Iwatsuki
- Renuka V Iyer
- Louise Izatt
- Grant Izmirlian
- Elena Jachetti
- Graham H Jackson
- Rene Jackstadt
- Francis Jacob
- Ritika Jaini
- Siddhartha Jaiswal
- Anders Jakobsen
- Nigel B Jamieson
- Tobias Janowitz
- MB Jarstfer
- James Java
- Michael Jeltsch
- Henry Jensen
- Seung-Yong Jeong
- Rinath Jeselsohn
- Jiafu Ji
- Aimin Jian
- Wenguo Jiang
- Xiaoyu Jiang
- Susan S Jick
- Duncan Jodrell
- Christoffer Johansen
- Tom John
- Mohit Kumar Jolly
- J Louise Jones
- Michael E Jones
- Robert Peter Jones
- Robin L Jones
- Joanna Jonska-Gmyrek
- Jingfang Ju
- Rudolph L Juliano
- Michaela Jung
- Sushant Kachhap
- Nina S Kadan-Lottick
- Kyuichi Kadota
- Christoph Kahlert
- Ulf D Kahlert
- Mihoko Kai
- Benny Kaipparettu
- Pawel Kalinski
- Steve E Kalloger
- Farin Kamangar
- Jian Kang
- Vinodh Kannappan
- Ellen Kapiteijn
- Dev Karan
- Anthony N Karnezis
- Antoine Karnoub
- Georg Karpel-Massler
- Ioannis Karydis
- Kozo Kataoka
- Jakob Kather
- Ken Kato
- Masaru Katoh
- Elad Katz
- Joonas H Kauppila
- Shinsuke Kazama
- Ai-Wu Ke
- Electron Kebebew
- Tom J Keegan
- Nancy E Kemeny
- Timothy Kendall
- Hirotsugu Kenmotsu
- Hagen Kennecke
- David Kerr
- Rebecca Kesselring
- Hector Charles Keun
- Alan Soo-Beng Khoo
- Sharon L Kilbreath
- Kyoung-Mee Kim
- Nam Kyu Kim
- Raymond H Kim
- Simon Kim
- Sung-Hoon Kim
- Tackhoon Kim
- Won Kim
- Alec C Kimmelman
- Hideharu Kimura
- Amy Ashley Kirkham
- Gaspar J Kitange
- Tobias Klatte
- Jorg Kleeff
- Alison Klein
- Suzanne Klimberg
- Carolyn Klinge
- Konrad Klinghammer
- Matthias Kloor
- Eugene J Koay
- Hiroki Kobayashi
- Raphael Koch
- Martin Koebel
- Maria Kokkinos
- Christian Kollmannsberger
- Shuhei Komatsu
- Stefan Kommoss
- Marcel Kool
- Miriam Koopman
- Michael I Koukourakis
- Jaco Kraan
- Onno Kranenburg
- Daniel Krappman
- Przemek Krawczyk
- Matthew Krebs
- Philippe Krebs
- Judith R Kroep
- Matthias Kroiss
- Ian E Krop
- Ilona Kryczek
- Kuan-Lin Kuo
- Charlotte Kuperwasser
- Ivana Kurelac
- Natasha Kyprianou
- Maria Kyrgiou
- Jon Amund Kyte
- Matthias Löhr
- Daniele La Forgia
- Valeria La Pietra
- Mark LaBarge
- SIntidhar Labidi-Galy
- M Lagios
- Barry J Laird
- Enzo Lalli
- Clara JK Lam
- David CL Lam
- Angela Lamarca
- Benjamin W Lamb
- Matteo Lambertini
- Claudia Lanari
- Ninian N Lang
- Cord Langner
- Ulrik Lassen
- Justin Lathia
- Ayse Latif
- Kirsten Lauber
- Kristina Laugesen
- Chris Laustsen
- James S Lawson
- Matt Lazzara
- Christophe Le Tourneau
- Estelle Leclerc
- Erinna F Lee
- Eunjung Lee
- Ho-Joon Lee
- Hye Seung Lee
- Jung-Min Lee
- Yunlong Lei
- Natasha Leighl
- Douglas A Levine
- MT Lewis
- Jin-ping Li
- Jingmei Li
- Tao Li
- Xiujuan Li
- Quan Liao
- Wen-Ting Liao
- Zhongxing Liao
- Jennifer Ligibel
- Jong-Seok Lim
- Sseak-Teak Lim
- Yi Chieh Lim
- De-Chen Lin
- Nancy U Lin
- Sun Linchong
- Michael Lind
- Claus Lindbjerg Andersen
- Jan Lindebjerg
- Kristina Lindemann
- Colin R Lindsay
- Michael Linnebacher
- Jeff Lipton
- Cheng Liu
- H Liu
- Na Liu
- Yongqing Liu
- Patrick Lizotte
- Antonio Llombart-Cussac
- Elena Lo Presti
- Fiona Lofts
- Sherene Loi
- Vinata B Lokeshwar
- Martijn Lolkema
- Valter Longo
- Charles Loprinzi
- Christopher J Lord
- Jonathan Loree
- Paul Lorigan
- Patricia M LoRusso
- Tamara L Lotan
- Fotios Loupakis
- Maeve A Lowery
- Noel Lowndes
- Tzu-Pin Lu
- Claudio Luchini
- Alessandro Lugli
- Sophia Lunt
- Guopei Luo
- Kongjia Luo
- XY Luo
- Rodney B Luwor
- Fiona Lyng
- Heidi Lyng
- Georgios Lyratzopoulos
- Volkmar Můller
- Yuk Ting Ma
- Teresa Macarulla
- Valentine M Macaulay
- Fergus Macbeth
- Zuzana Macek Macek Jilkova
- Thomas Mack
- Peter I Mackenzie
- Iain RJ Macpherson
- Yoshihiko Maehara
- Anthony M Magliocco
- Daruka Mahadevan
- Eamonn R Maher
- Ishrat Mahjabeen
- KM Mahoney
- Margarita Majem
- Umberto Malapelle
- Zvi Malik
- Dalvinder Mandair
- Nataly Manjarrez-Orduño
- Muhammad Mansha
- Janine L Mansi
- Anthony Maraveyas
- Caterina Marchiò
- Michael W Marcus
- Ofer Margalit
- Raphael Margueron
- Guillermo Mariño
- John M Mariadason
- Carmela Marino
- Angela Mariotto
- Aileen Marshall
- Agueda Martínez-Barriocanal
- Sarah Martin
- Miguel Martin-Caraballo
- Ubaldo Martinez-Outschoorn
- Tanimola Olanrewaju Martins
- Atsushi Masamune
- Giovanna Masci
- Chris Masden
- Christophe Massard
- Nader N Massarweh
- Shinobu Masuda
- Yohei Masugi
- Joaquin Mateo
- Aleyamma Mathew
- Kazumasa Matsumoto
- Charles E Matthews
- Julia Mayerle
- Clara Mayo de las Casas
- Neil McCarthy
- Valerie McCormack
- Keith McCrae
- Marjorie L McCullough
- Stuart Mcdonald
- Sean McGuire
- Ross McKinnon
- Úna McMenamin
- Donald C McMilllan
- Richard JQ McNally
- Sean McPhail
- Stephen T McSorley
- Benjamin Mead
- Vanina A Medina
- José Medina Echeverz
- Christoph R Meier
- Jane Meisel
- Alan A Melcher
- Sebastian Meltzer
- Javier A Menendez
- Bertrand Mennecier
- Rebecca Mercieca-Bebber
- Wilma E Mesker
- EA Mesri
- Meriem Messaoudene
- David Messenger
- Robert Lewin Metcalf
- Michael Michael
- Kenneth A Miles
- Linda R Mileshkin
- Lucia Miligi
- Anthony Bernard Miller
- Rowan Miller
- Todd W Miller
- Ken Mills
- Dragana Milojkovic
- Koshi Mimori
- Roberto Mina
- Anna Minchom
- Paul S Mischel
- Vivek Misra
- Akira Miyajima
- Yuu Miyauchi
- Jun Miyazaki
- Aviram Mizrachi
- Dominik P Modest
- Helmout Modjtahedi
- Siver A Moestue
- Hisham Mohammed
- Yejin Mok
- Alexander Molassiotis
- Damian J Mole
- David R Mole
- Remco J Molenaar
- Marie Monaco
- Debasis Mondal
- Clara Montagut
- Robert Montal
- Filippo Montemurro
- Susan L Mooberry
- Javier Mora
- Andrea Morandi
- Lluis Morey
- Martina Morini
- Karl Morten
- Juan-Miguel Mosquera
- Sophie Mouillet-Richard
- Kent W Mouw
- Yvonne Mowery
- Pavlos Msaouel
- David Mu
- Giridhar Mudduluru
- Alexander Muir
- Rebecca Muirhead
- Abhik Mukherjee
- William Mullen
- Patricia Muller
- Gabriele Multhoff
- Enrico Munari
- Gnanasekar Munirathinam
- Thijs Oude Munnink
- Jordi Muntane
- Yoshiki Murakumo
- Peter Murchie
- Daniel James Murphy
- Graeme I Murray
- Gokhan Mutlu
- Manabu Muto
- Bruna Muys
- Mette Nørgaard
- Ernest Nadal
- Michelle Nadler
- Gayathri Nagaraj
- Nico Nagelkerke
- Kalnisha Naidoo
- Rishi Naik
- Kiyotaka Nakano
- Sangkil Nam
- Steven A Narod
- Paul D Nathan
- Rachael Natrajan
- Atsushi Natsume
- Jean-Charles Nault
- Rudolph Navari
- Silvio Naviglio
- Sarah Nechuta
- Albrecht Neesse
- Joseph Neglia
- Eva Negri
- Kirsten Ness
- Daniel Nettersheim
- Polly Newcomb
- Edward M Newman
- Hazel B Nichols
- Simone P Niclou
- Chiara Nicolazzo
- Milena S Nicoloso
- Guenter Niegisch
- M Angela Nieto
- Kouki Nio
- Joanna Nixon
- Woo Chul Noh
- Jason Nomburg
- Norio Nonomura
- Helin V Norberg
- Lyse A Norian
- Sean M O’Cathail
- Grainne M O’Kane
- Ina Oehme
- Shuji Ogino
- Kadoaki Ohashi
- Shinji Ohno
- Karin A Oien
- Ichiro Okamoto
- Jan Oldenburg
- Sabrina Oliveira
- Francisco J Oliver
- Robert Olson
- Ian N Olver
- Christiane A Opitz
- Akira Orimo
- Clodia Osipo
- Harry Ostrer
- Quinn T Ostrom
- Ichiro Ota
- Christian Ottensmeier
- Thomas J Ow
- Tetsuhiro Owaki
- Masato Ozaka
- Belén P Solans
- Nicolle Packer
- Timothy P Padera
- Mark Pagel
- Claire Palles
- Carlo Palmieri
- Dario Palmieri
- Hardev S Pandha
- Klaus Pantel
- Demetris Papamichael
- James H Park
- Yong-Moon Park
- Amelia Parker
- Eileen Parkes
- Ramon Parsons
- Ann H Partridge
- Boris Pasche
- Oihana Pascual
- Silvia Pastorekova
- Alpa Patel
- Claire Paterson
- Maxime Patout
- Edward Pavlik
- Cloud Paweletz
- Michael Peake
- Alex Pearson
- Barrie Peck
- Stine Falsig Pedersen
- Rossella Pellegrino
- Juha Peltonen
- Majorette Pena
- George Pentheroudakis
- Carlos Perez-Plasencia
- Jonathan Perkins
- Claire M Perks
- Simon Pernot
- Aurélie Perraud
- Colin Perry
- Shazib Pervaiz
- Ljubica Petrovic
- David Pfister
- Paul D Pharoah
- Roger M Phillips
- Mike Philpott
- Amanda Phipps
- Alain Piché
- Martin Pichler
- Curtis Pickering
- Richard Piekarz
- Jean-Yves Pierga
- Géraldine Pignot
- Patrick Pilie
- Céline Pinheiro
- Paul F Pinsky
- Maria Elena Pisanu
- Barbara Pistilli
- Cameron Platell
- Stephen Plymate
- Katherine L Pogue-Geile
- Jan Poleszczuk
- Giuseppe Poli
- Andrew Polson
- Yves Pommier
- Mariano Ponz-Sarvise
- Camillo Porta
- James Posey
- Marshall Posner
- Sarka Pospisilova
- Pieter Edsge Postmus
- Chantevy Pou
- George Poulogiannis
- Simon N Powell
- Manohar Pradhan
- Prateek Prasanna
- Amy Prawira
- George Prendergast
- Timothy J Price
- Paul Proost
- Chamindie Punyadeera
- Kash Purohit
- Lisha Qi
- Wei-xiang Qi
- David Qian
- Max Qian
- Liang Qiao
- Yong Qin
- Andong Qiu
- Pietro Quaglino
- Christoph Röcken
- Curzio Růegg
- Joshua Rabinowitz
- Markus Raderer
- Jiannis Ragoussis
- Arjun Raj
- Christy Ralph
- Karthik Ramasamy
- Vijay Ramaswamy
- Joe Ramos
- Manreet Randhawa
- Anna Randi
- Atul Ranjan
- Sheela Rao
- Isabelle Ray-Coquard
- Daniel Rayson
- Anne Hansen Ree
- Mary E Reid
- Daniel Reimer
- Zachary J Reitman
- Dong Ren
- ShanCheng Ren
- Shengxiang Ren
- Michele Reni
- Daniel J Renouf
- Cristina Renzi
- Rodolfo Rey
- C Patrick Reynolds
- John V Reynolds
- Steven Reynolds
- Nicole Rich
- Des R Richardson
- Jennifer R Rider
- Carsten Riether
- Lorenza Rimassa
- Anja Rinke
- Gail Risbridger
- Barbara Rivera
- Kathryn Robb
- Jacques Robert
- Fiona Roberts
- Erle S Robertson
- Bridget Robson
- Caio Max S Rocha-Lima
- Annie Rochette
- Andrew W Roddam
- Rene Rodriguez
- Sandra Rodriguez-Perales
- Achim Rody
- Casper Roed
- Paola Romagnani
- Eve Roman
- Andrea Romano
- Lisa M Rooper
- Rafael Rosell
- Kirill V Rosen
- Remi Rosiere
- Tania Roskams
- Paul G Rothberg
- Varda Rotter
- Montserrat Rovira
- Campbell SD Roxburgh
- Patricia Roxburgh
- Damjana Rozman
- Greg Rubin
- T Russell
- Mark J Rutherford
- Elizabeth J Ryan
- Francisca Sánchez Jiménez
- Mart Saarma
- Renaud Sabatier
- Joseph Sacco
- Anguraj Sadanandam
- Hiroshi Saeki
- Sumit Sahni
- Jun Sakakibara-konishi
- Ramon Salazar
- Shahram Salek-Ardakani
- Jami Saloman
- David S Salomon
- Vassiliki Saloura
- Manuel Salto-Tellez
- Ashok Saluja
- Adel Samson
- Leslie Samuel
- Ian Sanders
- Vlad Sandulache
- Bruno Sangro
- Owen J Sansom
- Cristina Santos
- Deborah Saraggi
- Devanand Sarkar
- Agata Sasor
- Kazuhide Sato
- Mark P Saunders
- Philippa TK Saunders
- Stefania Scala
- Maurizio Scaltriti
- Sebastian Schölch
- Zsuzsa Schaff
- Robin Schaffar
- Tobias Schatton
- Daniel Schauer
- Joellen M Schildkraut
- Gudrun Schleiermacher
- Barbara Schmalfeldt
- Annina Schmid
- Manuela Schmidinger
- Kjeld Schmiegelow
- Regine Schneider-Stock
- Minouk J Schoemaker
- Sara Schonfeld
- Zachary T Schug
- Almut Schulze
- Albrecht Schwab
- Marc D Schwartz
- Heidi Schwarzenbach
- Marco Sciacovelli
- Jean-Yves Scoazec
- David Scott
- Nina Scott
- Michael J Seckl
- Jan Sedlak
- John Paul Seenan
- Shanly C Seferina
- Andrei Seluanov
- Andrzej Semczuk
- Gregg L Semenza
- Toni T Seppälä
- Natalie Serkova
- Lucia Sfondrini
- Michael Shackcloth
- Erin Shanahan
- Chunlin Shao
- Rong Shao
- Zhi-Ming Shao
- Rohini Sharma
- Sherven Sharma
- Adam Sharp
- Alice Shaw
- M Shekhar
- Cheng-Che Shen
- Jie Shen
- Li Shen
- Wen Shen
- Qiuling Shi
- Yiwey Shieh
- Anthony F Shields
- Meredith Shiels
- Alan Shih
- Akihiko Shimomura
- Eiji Shinto
- Tanri Shiozawa
- Brian Shirts
- Haim Shmuely
- Alexander Noor Shoushtari
- Christina Signorelli
- Abigail Silva
- Andrew Silver
- Camelia Sima
- Kate Simms
- Ronald Simon
- Ajay Pratap Singh
- Pankaj Singh
- Rakesh K Singh
- Sharad S Singhal
- Hans-Peter Sinn
- Tobias Sinnberg
- Peter J Siska
- Nils Skajaa
- Rodrick Skinner
- Margaretha Skowron
- Zdenek Skrott
- Laurence Slembrouck
- Joseph Slupsky
- Grace Li Smith
- Graeme Smith
- Robert Smith
- Elizabeth C Smyth
- Claire Snyder
- Eric Snyder
- Li-bing Song
- Liqing Song
- Mingyang Song
- Gabe S Sonke
- Juergen Sonnemann
- Pierre Sonveaux
- Ashwani Sood
- Julio Sotelo
- Federica Sotgia
- Margarida Souto-carneiro
- Giulio Spagnoli
- Paul Span
- Joseph A Sparano
- Logan G Spector
- James F Spicer
- Michael J Spinella
- Henrik Støvring
- Steven A Stacker
- Martin Stanulla
- Justin Stebbing
- Susan E Steck
- Andrew Steele
- Stacey M Stein
- Bettie M Steinberg
- Leif Stenke
- Herbert Stepp
- Lindsay Stetson
- Grant Stewart
- Paul Stewart
- Sherri Stewart
- Christine Stoehr
- Konrad Stopsack
- Phillip D Stricker
- Amit Sud
- Kotaro Sugawara
- Yasutaka Sukawa
- Saraswati Sukumar
- A Sultan
- Haibo Sun
- Li-Min Sun
- Roger Sun
- Shuyang Sun
- Yihua Sun
- Yu Sun
- Karin Sundström
- Max W Sung
- Ruth Swann
- David Swanson
- Randy Sweis
- Anthony J Swerdlow
- Daniel Swinson
- Matt Sydes
- Peter Wojciech Szlosarek
- Alphonse G Taghian
- Julien Taieb
- Naruto Taira
- Hidenori Takahashi
- Toshimi Takano
- Koji Takeda
- Valerie Taly
- David Tan
- Tuan Zea Tan
- Shinji Tanaka
- Shota Tanaka
- Torgrim Tandstad
- Menghua Tao
- Yongguang Tao
- Mehmet Taspinar
- Daniele Tauriello
- JA Taylor
- Andrew R Tee
- Chung-Jen Teng
- Daniel A Tennant
- Theodosia Teo
- Masanori Terashima
- M Terzolo
- Christina Therkildsen
- Chrissie Thirlwell
- Liz Thompson
- Fiona Thomson
- Andrew Thorburn
- Jeanne Tie
- Daniel Guimaraes Tiezzi
- Wayne D Tilley
- Ingeborg Tinhofer
- Anna Tinker
- Herve Tiriac
- Marc Tischkowitz
- Adriane Regina Todeschini
- V Todorova
- Robert Toillon
- Erik Tokar
- Alex Toker
- Ian PM Tomlinson
- Giuseppe Tonini
- Davis Y Torrejon
- Valter Torri
- Michael Toss
- Jorg Tost
- Christos Toumpanakis
- Rhian Touyz
- Amanda R Townsend
- Gouji Toyokawa
- Lois B Travis
- Sophie Trefely
- Edward L Trimble
- Isabelle Trouilloud
- Dean Troyer
- Takemasa Tsuji
- Musaffe Tuna
- Shannon Turley
- Chris Twelves
- Hiroyuki Uetake
- Henrik Ugge
- Sang-Won Um
- Viktor Umansky
- Renato Umeton
- Craig Underhill
- Ravindra Uppaluri
- Kevin Y Urayama
- Jay V Vadgama
- Pierre Val
- Fatima Valdes-Mora
- Nicola Valeri
- Jeff Vallance
- Laura Valle Velasco
- Benoit Van den Eynde
- Antoine G van der Heijden
- Gabri van der Pluijm
- Casper HJ van Eijck
- Folkert J Van Kemenade
- Sandra Van Schaeybroeck
- Pieter van Vlierberghe
- Sakari Vanharanta
- Peter Vedsted
- Sergia Velho
- Balaji Venugopal
- Pasquale Verde
- Henk M Verheul
- Louis Vermeulen
- Chris Verslype
- Jan Vijg
- Bruno Vincenzi
- Igor Vivanco
- Ana Vivancos
- Arndt Vogel
- Elena Voronov
- Madhuri Wadehra
- Derek Wainwright
- Michael Wakelam
- Lucy Wall
- K Wallace
- Jo Waller
- Harriet Walter
- Fubo Wang
- Greg Wang
- Jian-Bing Wang
- Kai Wang
- Michael Wang
- Y Wang
- Yi-Ching Wang
- Ying-Jan Wang
- Yong-Sheng Wang
- Zhenning Wang
- Zhigang Wang
- Zhijie Wang
- Zhiwei Peter Wang
- Miriam Wanner
- Fredrik Warnberg
- Ellen Warner
- David Warr
- James Wason
- Justin Waters
- Malcolm Watford
- Randolph Watnick
- David C Wedge
- Jon Wee
- Daoyan Wei
- Jean M Weigert
- Benjamin Weinberg
- Paul Weinberger
- Joachim Weischenfeldt
- Noel S Weiss
- Stephen J Weiss
- Alan Wells
- James Welsh
- Michael Welsh
- Bruce Wenig
- Haim Werner
- Finn Wesenberg
- Mike-Andrew Westhoff
- William Westra
- Jeff D White
- Richard White
- James Whiteford
- Theresa L Whiteside
- Malin Wickstrom
- Martin Widschwendter
- Stefan Wiemann
- Joseph Wiemels
- Lisa Wiesmueller
- Fredrik Wiklund
- Gareth Haydn Williams
- Vernell Williams
- Brian C Wilson
- Christina Wilson
- William R Wilson
- Simon Wing
- Renate Winkels
- John Winslow
- Jorg Wischhusen
- Ken Witwer
- Solomon Woldu
- Klaus-Dietrich Wolff
- Monika Wolkers
- Michael O Woods
- Sir Nick Wright
- Ludovic Wrobel
- Erxi Wu
- Haili Wu
- Victor Wunsch Filho
- Hongqing Xi
- Tian-Song Xia
- Jianbin Xiang
- Shuai Xiao
- Dongqing Xu
- Liang Xu
- Yong-Bin Yan
- Haining Yang
- Xiaoyi Yang
- Yaohua Yang
- James C Yao
- Tso-Pang Yao
- Christina Yap
- Koichiro Yasaka
- Masakazu Yashiro
- Weimin Ye
- Nelson Yee
- Rinat Yerushalmi
- Min Yi
- Y Yilmaz
- Xiao-Ming Yin
- Kent Yip
- Gregory S Yochum
- Yongping You
- Heather Young
- Susan Yount
- Ke-Da Yu
- Kenneth H Yu
- Tung-Min Yu
- Xian-Jun Yu
- Xuefeng Yu
- Fiona Yull
- Gérard Zalcman
- Mattia Zampieri
- Maurizio Zanetti Zanetti
- Paul Zarogoulidis
- AG Zeimet
- Hitoshi Zembutsu
- Hao Zeng
- Kaixuan Zeng
- Olga Zeni
- Ioannis Zervantonakis
- Yiqiang Zhan
- Donna Zhang
- Guodong Zhang
- Hongquan Zhang
- Jian Zhang
- Lei Zhang
- Ning Zhang
- Shawn (Xiang) Zhang
- Tai-Ping Zhang
- Xiaotun Zhang
- Yi Zhang
- Zhen Zhang
- Zhongyin Zhang
- Jian-Yuan Zhao
- Senlin Zhao
- Xiaodi Zhao
- Lufeng Zheng
- Jian-hong Zhong
- Haijun Zhou
- Hongyu Zhou
- Zizhang Zhou
- Andries Zijlstra
- Manuel Zorzi
- Zhi-Peng Zou
- Jan Zuna

